# A Large Ensemble Global Dataset for Climate Impact Assessments

**DOI:** 10.1038/s41597-023-02708-9

**Published:** 2023-11-14

**Authors:** Xiang Gao, Andrei Sokolov, C. Adam Schlosser

**Affiliations:** MIT Joint Program on the Science and Policy of Global Change, Cambridge, MA 02139 USA

**Keywords:** Climate sciences, Climate-change impacts

## Abstract

We present a self-consistent, large ensemble, high-resolution global dataset of long‐term future climate, which accounts for the uncertainty in climate system response to anthropogenic emissions of greenhouse gases and in geographical patterns of climate change. The dataset is developed by applying an integrated spatial disaggregation (SD) − bias-correction (BC) method to climate projections from the MIT Integrated Global System Model (IGSM). Four emission scenarios are considered that represent energy and environmental policies and commitments of potential future pathways, namely, Reference, Paris Forever, Paris 2 °C and Paris 1.5 °C. The dataset contains nine key meteorological variables on a monthly scale from 2021 to 2100 at a spatial resolution of 0.5°x 0.5°, including precipitation, air temperature (mean, minimum and maximum), near-surface wind speed, shortwave and longwave radiation, specific humidity, and relative humidity. We demonstrate the dataset’s ability to represent climate-change responses across various regions of the globe. This dataset can be used to support regional-scale climate-related impact assessments of risk across different applications that include hydropower, water resources, ecosystem, agriculture, and sustainable development.

## Background & Summary

Global climate model simulations of the past, current, and future climate have become readily available through different phases of the Coupled Model Intercomparison Project (CMIP). Over the past several decades, the CMIP data archive has served as a foundational element of climate science and for supporting decision and policy-makers communities. The recent sixth phase of CMIP (CMIP6) has expanded the breadth of the coordinated climate model experiments considerably with more than 70 participating models of a wide variety from more than 30 participating model groups worldwide^1^.

The Scenario Model Intercomparison Project (ScenarioMIP) in CMIP6 provides the main set of future climate projections based on alternative scenarios directly relevant to societal concerns for climate change mitigation, adaptation, or impacts^2^. These climate projections are driven by a set of emissions and land use scenarios produced with integrated assessment models (IAMs) based on future pathways of societal development, the Shared Socioeconomic Pathways (SSPs), and related to the forcing levels of the Representative Concentration Pathways (RCPs)^3^. Compared to CMIP5, CMIP6 RCPs fill critical gaps for intermediate forcing levels (for example, short-lived species and land use) and the full set of SSPs-RCPs combinations spans a larger range of outcomes. However, these multi-model climate projections under a matrix of possible integrated scenarios may not adequately represent the desired risk-based assessment of climate change impacts and climate policy benefits due to several issues described below. First, there is a lack of consistency in socioeconomic and environmental factors and such consistency is highly relevant for assessing climate impacts and climate policy benefits. The CMIP model projections are exogenously driven via greenhouse gas concentrations, and therefore do not account for the direct responses and linkages between natural processes and human emissions and other climate-relevant activities. Further inconsistencies are attributed to the disconnected users and developers that develop the shared socioeconomic scenarios, and thus lead to difficulties in interpreting different model results, despite a common implemented scenario target (e.g., land use). Further, the collection of CMIP6 SSP-RCP scenarios was not produced by one IAM, but rather from a selection of distinctly different IAMs developed at institutes around the world. The emission and socioeconomic projections generated from these IAM scenarios are often not consistent with one another and could thus compromise any cross-scenario comparative analysis of climate policy benefits, climate-related impacts, transition risks, and sustainable development consequences. Second, global climate model outputs are prone to errors/biases that can be caused by a range of factors, including limited spatial resolution (large grid sizes), simplified thermodynamic processes and physics or incomplete understanding of the global climate system. The use of biased climate model outputs in impact models (i.e., crop models for agriculture, water resource models for hydrology) or local-scale climate impact assessment can often lead to unrealistic and distorted results. Third, the ensemble size of projections from the participating climate/Earth-system models are limited in providing quantitative insights on ‘risk’ or the probability of variables of interest.

We address these issues by developing a self-consistent, large ensemble, high-resolution, bias-corrected global dataset of future climates for a range of global emission pathways and/or climate targets. The dataset can be viewed as an update and commensurate to similar products based on earlier versions of CMIP^4^ and meets the data needs of studies on regional, risk-based climate change impacts across different applications, such as ecosystem^5,6^, agriculture^7,8^, water resource^9–11^, hydropower^12^, environment^13^, and sustainable development^14^. Our method combines plausible patterns of human-forced regional climate change with a comprehensive characterization of the global climate change response as determined by the MIT Integrated Global System Model (IGSM)^15^. IGSM consists of the linked MIT Earth System Model (MESM) of intermediate complexity and the Economic Projections and Policy Analysis model (EPPA) for analyzing human-Earth systems interactions^15^. The EPPA characterizes detailed economic activities to track inter-sectoral and inter-regional links, while the MESM represents key physical, chemical, and biological components of the Earth system that are impacted by human activity. Such integrated framework ensures consistent treatment of interactions among population growth, economic development, energy and land system changes and physical climate responses, which can provide improved assessments of climate impacts across multiple sectors^16^. The MESM contains a two-dimensional (zonally averaged, explicit representation of latitude and altitude) atmospheric model with interactive chemistry coupled to the zonally averaged version of Global Land System model and an anomaly-diffusing ocean model. This architecture allows for conducting a large ensemble of climate simulations for robust uncertainty analyses at significantly less computational cost than state-of-the-art climate models. In particular, the parameters that characterize climate sensitivity, the rate of ocean heat uptake, and the strength of aerosol forcing can be varied in MESM, a powerful feature to explore a range of uncertainty in the climate response. We first carry out a 50-member ensemble of MESM simulations (zonal) across a range of global emission pathways produced by EPPA. We then apply a combined spatial disaggregation (SD) – bias correction (BC) method. SD uses a pattern-scaling approach to achieve longitude representation at the desirable spatial resolution, while BC corrects the inherent biases in the MESM future climate projections.

## Methods

### Data acquisition

Various datasets used in this study and their characteristics are summarized in Table 1 and detailed as below.

**Table 1:** Various datasets used in this study and their characteristics.

Data (ensemble)	Temporal (Span)	Spatial (domain)	Usage (data period)
GSWP3 (1)	3-hourly (1901–2014)	0.5° × 0.5° (global)	baseline climate (1931–2010) and historical pattern-scaling kernels ${\bar{C}}_{xy}^{{t}_{0}}$(1991–2010)
MESM (50)	monthly (1861–2100)	46 zonal bands (44 4° bands and 2 2° bands near both poles, global)	delta change (1991–2010 for ${\bar{V}}_{y}^{{t}_{0}}$, 2002–2100* for ${\bar{V}}_{y}$, and 2012–2100* for *Δ*${\bar{T}}_{G}$)
CMIP6 1pctCO_2_ (18)	monthly (variable)	variable (global)	pattern-changing kernels (1^st^ ~ 10^th^ and 71^th^ ~ 80^th^ years)

*These periods include the years for calculation of moving averages. The resulting delta changes span from 2021–2100.

#### Historical meteorology

We utilize the historical meteorological dataset from the third phase of the Global Soil Wetness Project (GSWP3, http://hydro.iis.u-tokyo.ac.jp/GSWP3/) as the baseline climate. It is a 3-hourly 0.5° global forcing product (1901–2014) based on the 20^th^ Century Reanalysis version 2^17^. The reanalysis was dynamically downscaled to 0.5° resolution based on the Global Spectral Model using a spectral nudging technique^18^. Bias corrections based on observations were made for temperature, precipitation, longwave radiation, and shortwave radiation using CRU TS v3.21 (Climate Research Unit)^19^, GPCCv7 (Global Precipitation Climatology Centre)^20^, and Surface Radiation Budget data sets, respectively. A wind-induced undercatch correction was also applied.

The meteorological variables considered in this study include precipitation, air temperature (mean, minimum and maximum), near-surface wind speed, shortwave and longwave radiation, specific humidity, and relative humidity. Minimum and maximum air temperature as well as relative humidity are not provided in the GSWP3 data, but derived as follows. Minimum and maximum air temperature are calculated as the monthly means of the minimum and maximum daily air temperature across the 8 3-hourly time steps, respectively. Relatively humidity is derived from surface air temperature (*T*_*air*_), surface air pressure (*Psf*), and specific humidity (*Qair*) at a 3-hourly time step as in Eq. (1) and averaged into the monthly values.

1 $${e}_{s}=6.112\ast {e}^{\frac{17.67\ast {T}_{air}}{{T}_{air}+243.5}},e={Q}_{air}\ast \frac{Psf}{0.378\ast {Q}_{air}+0.622},rh=100\ast \frac{e}{{e}_{s}}$$

Where *e* and *e*_*s*_ are vapor pressure and saturated vapor pressure, respectively. *rh* is the relative humidity in percent.

### CMIP6 model simulations

The CMIP has become one of the foundational elements of climate science by distributing global climate model simulations of the past, current, and future climate. The CMIP6 includes more than 70 participating models of a wide variety developed at research institutes across the international scientific community. For the SD pattern-scaling procedure used in the construction of our large-ensemble future projections at the 0.5° resolution, we draw the simulations of the participating models from the 1pctCO_2_ experiment in which the concentration of atmospheric CO_2_ increases gradually at a rate of 1% per year from the global annual mean 1850 value until quadrupling. It serves as a consistent and useful benchmark for analyzing model transient climate response (TCR) to cumulative carbon emissions (TCRE). Our prior published work^21^ showed that similar response patterns are obtained for a particular climate model, regardless of the CMIP scenarios (i.e., 2xCO_2_ and RCPs), suggesting that the patterns are robust and represent a consistent forced response due to the human-induced radiative forcing. Multiple variations of model outputs may be available from a model family with different configurations (e.g., different spatial resolutions or components). In that case, only one sibling model is selected to represent the family. The precedence for selection is (highest to lowest): (1) all the key meteorological variables of our interest are archived, including precipitation, air temperature (mean, minimum and maximum), near-surface wind speed, shortwave and longwave radiation, specific humidity, and relative humidity; (2) an Earth System Model (ESM) is chosen whenever available; and (3) higher spatial resolution is desired. This resulted in 18 climate models that participated in the CMIP6 1pctCO_2_ experiment (Table 2). Their corresponding monthly outputs are then obtained for the pattern-scaling method (Section 3.2).

**Table 2:** List of CMIP6 models used in our study to construct the pattern-scaling kernels of climate change response.

Model Name	Resolution	Institution
ACCESS-ESM1-5	1.875° × 1.25°	Australian Commonwealth Scientific and Industrial Research Organization
AWI-ESM-1-1-LR	1.875° × 1.875°	German Alfred Wegener Institute
BCC-CSM2-MR	1.125° × 1.125°	Beijing Climate Center
CanESM5	2.8125° × 2.8125°	Canadian Centre for Climate Modelling and Analysis
CMCC-ESM2	1.25° × 0.9375°	Centro Euro-Mediterraneo Cambiamenti Climatici
CNRM-ESM2-1	1.40625° × 1.40625°	Centre National de Recherches Meteorologiques
EC-Earth3-Veg	0.703125° × 703125°	EC-Earth-Consortium
FGOALS-g3	2.0° × 2.25°	Chinese Academy of Sciences
FIO-ESM-2-0	1.25° × 0.9375°	Qingdao National Laboratory for Marine Science and Technology
GISS-E2-2-G	2.5° × 2.0°	Goddard Institute for Space Studies
HadGEM3-GC31-MM	0.83° × 0.56°	Met Office Hadley Centre
INM-CM5-0	2.0° × 1.5°	Russian Academy of Science
IPSL-CM6A-LR	2.5° × 1.26°	Institut Pierre Simon Laplace
MIROC-ES2L	2.8125° × 2.8125°	Japan Agency for Marine-Earth Science and Technology
MPI-ESM1-2-HR	0.9375° × 0.9375°	Max Planck Institute for Meteorology
MRI-ESM2-0	1.125° × 1.125°	Japan Meteorological Research Institute
SAM0-UNICON	1.25° × 0.9375°	Seoul National University
UKESM1-0-LL	1.875° × 1.25°	Met Office Hadley Centre

#### MESM future climate projections

We focus on four policy scenarios that were developed to span a range of possible global actions to abate greenhouse gas emissions over the coming century, specifically **Reference (REF),** **Paris Forever (PF),** **Paris 2 °C (P2C)**, and **Paris 1.5 °C (P1p5C)**^11^. These policy scenarios are more aligned with the global emission pathways and/or climate targets established in the recent Paris Agreement. The REF scenario implements no explicit climate mitigation policy (no specific greenhouse gas emissions target) anywhere in the world for the sake of abating climate change, except for some energy policies such as fuel economy standards, renewable electricity requirements, the gradual phase-out of old coal power plants, reduced use of exhaustible resources, and conventional pollutants reduction. The REF serves as a baseline scenario and represents the upper bound of climate change risks. The PF scenario assumes that all Paris Agreement Nationally Determined Contributions (NDCs) are implemented through the year 2030 and maintained in perpetuity after that. While our PF scenario represents an unprecedented global commitment to limit greenhouse gas emissions, it neither stabilizes climate nor limits climate change. P2C and P1p5C scenarios extend from the Paris Agreement’s NDCs and align with its long-term goals. These two scenarios aim to limit and stabilize human-induced global climate warming to 2 °C and 1.5 °C, respectively, by the end of this century. Variations in mitigation policies lead to the uncertainty in patterns of resource and energy use, technology choices, and drag on overall economic growth, with additional uncertainty arising from the global climate response represented in the MESM^4^. These co-evolving uncertainties result in an overall probability of achieving the target at 50% for both P2C and P1p5C scenarios.

**Fig. 1:**
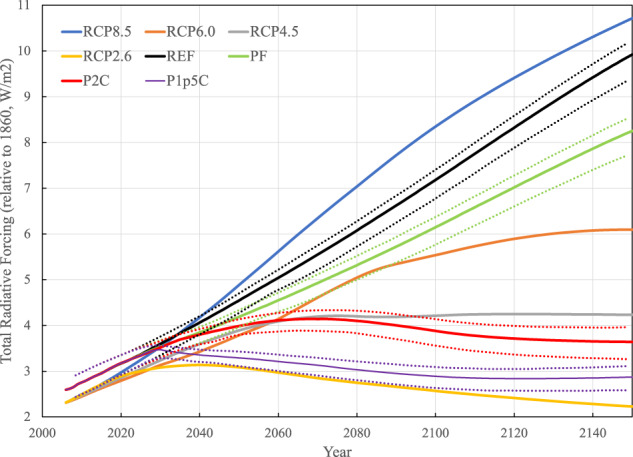
Total radiative forcing (relative to 1860, W/m^2^) from the four MESM policy scenarios used in this study, compared to that from the Representative Concentration Pathway (RCP) experiments in CMIP5. The dotted lines indicate 5% and 95% percentiles, respectively.

**Fig. 2:**
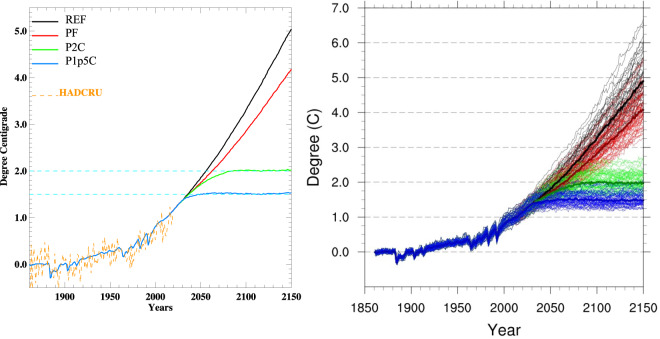
Global averaged annual surface-air temperature trends for the four MESM policy scenarios. The left panel presents the median trajectories of the 50-member MESM ensemble, while the right panel provides the trajectories from all ensemble members. Trends in temperature are calculated relative to the 1861–1880 mean.

The most recent version of MESM has been extensively evaluated^15^. Its performance against observations at the global and zonal scales is comparable to global climate models from CMIP5. As such, MESM is an efficient model that can faithfully produce the global- and zonal-scale plausible climate responses to anthropogenic drivers. For each of four policy scenarios, we carry out a 50-member ensemble of MESM simulations (zonal) to account for the uncertainty in the climate system response to anthropogenic emissions of greenhouse gases. These ensemble simulations are conducted using different values of model parameters that characterize climate sensitivity, rate of heat and carbon uptake by the ocean, and strength of aerosol forcing. 50 sets of these parameters are generated by Latin Hypercube sampling from 3-dimensional parameter distribution, which is obtained by comparing with observations the changes in surface temperature and deep ocean heat content simulated by MESM forced by historical forcing^22^. We find the simulated surface air temperature distribution based on this 50-member ensemble replicates well that based on the 400-member ensemble used in the 2021 Global Change Outlook^11^. These four policy scenarios depict distinct pathways and distributions of global averaged changes in human forcing and climate variables (Figs. 1, 2). In comparison with the CMIP5 RCP scenarios, the MESM REF scenario presents weaker radiative forcing than its RCP counterpart (RCP8.5), while the more aggressive climate-based targets (P2C and P1p5C) fall between RCP4.5 and RCP2.6 scenarios. The PF scenario characterizes stronger radiative forcing than the RCP6.0 scenario. The impact of the aggressive mitigation policies on alleviating or eliminating the warming risk is immediately evident, with a majority of the distributions of global averaged annual surface-air temperature (SAT) trends in P2C and P1p5C scenarios falling outside that of the REF scenario in the latter half of the 21^st^ century (Fig. 2). Despite a notable shift toward lower warming risk, considerable overlap persists till the end of the century between the SAT trends distributions of the PF scenario and those of the REF scenario.

### SD-BC method

We employ an integrated SD-BC method to develop a long-term (2021–2100), large ensemble, high-resolution global dataset of future climate projections. SD uses a pattern-scaling approach to transform the MESM zonal climate to longitude representation at the spatial resolution consistent with that of observations. BC methods rely on the use of different statistical techniques to make climate model outputs more realistic and also achieve finer spatial resolution if high-resolution observations are available^23,24^. BC can be implemented in various ways, including delta change, quantile mapping, linear regression, variance scaling, etc. The delta change method is the simplest approach and involves applying a changing factor to the historical observation for constructing a new time series of the future climate^4,24^. A changing factor can be additive (difference) or multiplicative (ratio). Different changing factors can be employed for different climate variables. This method does not take into account changes in climate variability such as increasing extreme rainfall or longer dry spells. Linear regression performs a regression analysis using historical observations and climate model outputs during the same period and applies the regression parameters to construct bias-adjusted future climate time series. The regression can be made as simple or complex^25^. Quantile mapping generally uses a Gaussian or gamma distribution function to correct the distribution function of a climate variable and improve its fitting to observations^26,27^. Quantile mapping has become widely used because of its ability to correct bias at the extreme tails and its desired accuracy and robustness. However, the results may be sensitive to the choice of calibration period. All these BC methods are univariate which correct one variable at a time. Some multivariate BC (MBC) methods have been recently proposed to account for inter-variable, spatial or temporal dependencies of the observations^28^.

Here we use the delta change method as our BC method. Despite its simpleness, the delta change method is still one of the popular methods for climate model bias correction^4^. Recent studies^29–31^ have shown that the delta change method performs as well as or occasionally better than complex bias correction techniques (i.e., generalized additive models (GAMs), quantile mapping). The low computational demand of the delta method facilitates an efficient bias-correction of MESM future climate across a large ensemble and diverse emission scenarios over centuries. The basic principle behind the BC delta method involves adding changes (or deltas) in projected monthly climate to the high-resolution present-day climate (baseline). In our study, baseline climate is constructed to be free of any significant monotonic trends caused by human forcing, while changes (deltas) represent only the human-forced monotonic trends from MESM’s global climate response (without internal climate variability). The projected monthly climate from the MESM is at the coarse resolution (zonal) and need be spatially disaggregated through a pattern-scaling technique (described below) to match the high-resolution baseline climate before the addition is applied. The delta method assumes that changes in climates are only relevant at coarse scales but temporal variability of each variable from the baseline climate is maintained towards the future. This assumption may hold true in many cases, except for the highly heterogeneous landscapes where considerable variations may be induced within a relatively short distance. The bias-corrected climate projections resulting from the delta change method could be negative if future trends of meteorological variables (changes or deltas) are negative and larger than the corresponding detrended baseline values. In our procedure, we simply mask out any negative value (reassigned as zero) to ensure data validity.

**Fig. 3:**
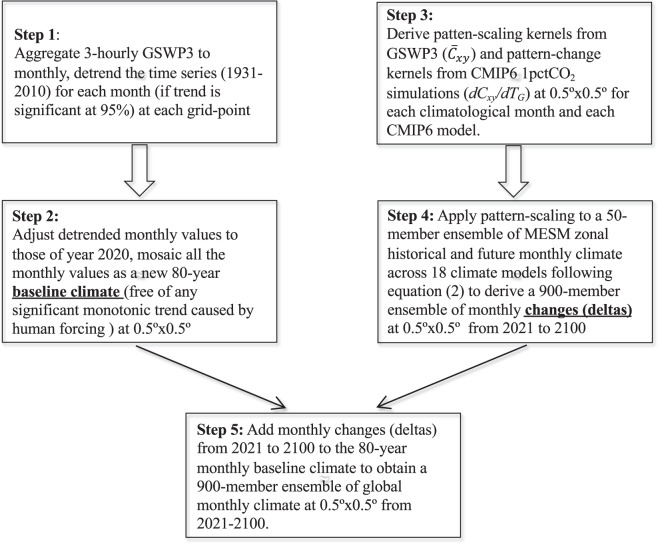
A schematic of the SD-BC method for producing future climate.

The diagram for the SD-BC method is shown in Fig. 3 and comprises the following steps: (1) detrending monthly historical climate time series (GSWP3, 1931–2010) for each month (if trend is significant at 5%) at each grid-point; (2) adjusting detrended monthly values to those of year 2020 and mosaicking all the monthly values as a new 80-year baseline climate (free of any significant monotonic trend caused by human forcing) at 0.5° × 0.5°; (3) deriving pattern-scaling kernels from GSWP3 and pattern-change kernels from CMIP6 1pctCO_2_ simulations at 0.5° × 0.5° for each climatological month and each CMIP6 model; (4) applying pattern-scaling to a 50-member ensemble of MESM zonal historical and future monthly climate across 18 climate models (Table 2) following Eq. (2)^21^ (described in pattern-scaling method) to derive a 900-member ensemble of monthly changes (deltas) at 0.5° × 0.5° from 2021 to 2100; and (5) adding monthly changes (deltas) from 2021 to 2100 in step 4) to the 80-year monthly baseline climate in step 2 to obtain a 900-member ensemble of global monthly climate at 0.5° × 0.5° from 2021–2100. The procedure is implemented for each meteorological variable and each emission scenario. Details for each step are elaborated below.

#### Detrended baseline climate

The essence of our SD-BC method is to obtain a baseline climate that is free of any significant monotonic trends (caused by human forcing) while preserving the variance/variability around the trend. We perform significance test for the linear trend of 80-year (1931–2010) GSWP3 monthly time series for each month at each grid-point. If a linear trend is found statistically significant at the 95% confidence level, the new monthly time series are derived by first detrending the original monthly time series and then adjusting (adding) the detrended monthly values to the corresponding monthly values of year 2020 to emulate an 80-year (2021–2100) climate without climate change. The monthly values of year 2020 are obtained based on the linear trend. If a linear trend is found not statistically significant, the new monthly time series will simply take the original monthly time series. The derived new monthly time series of all the months are then merged, which is referred to as “detrended baseline climate” in the following steps. For precipitation, we apply the trend significance test to the logarithm of monthly precipitation to account for the log-normal nature of precipitation^32^ with the underlying assumption of normality in the trend significance test. A linear relationship is examined only if at least half of the total years (80) have the pre-transformed monthly precipitation larger than 1 mm/day. This condition will prevent any artificial linear trend as a result of very low precipitation amount in very dry areas (e.g., Sahara Desert), which could lead to unrealistically high values in the detrended precipitation time series. Detrending precipitation (if a linear trend is significant) is only applied to the years that involve in constructing the linear relationship. Other years will simply take the original monthly values. Selective grid-by-grid detrending may introduce some level of spatial inconsistencies. We have tested the uniform grid-by-grid detrending (regardless of whether trends are determined to be significant or not) and found that it could lead to spurious, unrealistic extreme values (high and low). These artifacts arise mainly from imposing a linear relationship on an insignificant-trend time series and we thus must apply arbitrary thresholds to ensure that a reasonable range is maintained for each variable. The mosaicked detrended baseline is shown to not present salient spatial inconsistency but capture the expected large-scale climate features across various regions of the globe.

#### Pattern-scaling method

In climate-change scenario assessments and impact studies, the use of various pattern-scaling methods is extensive^33–38^. For the 50-member ensemble of the MESM zonal climate projections, we adopt a similar method to expand across longitudes using pattern-scaling specifically tailored to the MESM zonal configuration to provide regional representation^21^. The inception of this method for use with MESM and IGSM is described in^21^. The procedure is analytically expressed by the following equations:

2 $${\bar{V}}_{x,y}^{{t}_{0}}={\bar{C}}_{x,y}^{{t}_{0}}\ast {\bar{V}}_{y}^{{t}_{0}},{V}_{x,y}=\left({\bar{C}}_{x,y}^{{t}_{0}}+\left[\frac{d{\bar{C}}_{x,y}}{d{\bar{T}}_{G}}\ast \Delta {\bar{T}}_{G}\right]\right)\ast {\bar{V}}_{y}$$

3 $$\frac{d{\bar{C}}_{x,y}}{d{\bar{T}}_{G}}=\frac{{\bar{C}}_{x,y}^{{t}_{2}}-{\bar{C}}_{x,y}^{{t}_{1}}}{{\bar{T}}_{Global}^{{t}_{2}}-{\bar{T}}_{Global}^{{t}_{1}}}$$

Where the overbar represents the average value during reference periods (i.e., *t*_*0*_, *t*_*1*_, and *t*_*2*_) or a moving average period. ${\bar{C}}_{x,y}$ is climatological pattern-scaling coefficient and calculated as the ratio of monthly climatology at each grid-point to monthly climatology of the corresponding latitude mean $\left({\bar{V}}_{y}\right)$. ${\bar{C}}_{x,y}^{{t}_{0}}$ and ${\bar{V}}_{y}^{{t}_{0}}$ are derived from the 20-year (1991–2010, *t*_*0*_) GSWP3 data. ${\bar{V}}_{x,y}^{{t}_{0}}$ is the downscaled monthly climatology at a grid point (*x,y*) during the reference period *t*_*0*_. The choice of t_1_ and t_2_ is somewhat arbitrary but should span a sufficient amount of time such that a climate response (if any) has evolved as a result of the trends in the trace gas forcing. For the scenario considered herein, we chose the interval between *t*_1_ and *t*_2_ to span the number of years at which a doubling of CO_2_ at a transient rate of 1% yr^−1^ has been achieved, equivalent to 70 years. ${\bar{C}}_{x,y}^{{t}_{1}}$ $\left({\bar{T}}_{Global}^{{t}_{1}}\right)$ and ${\bar{C}}_{x,y}^{{t}_{2}}$ $\left({\bar{T}}_{Global}^{{t}_{2}}\right)$ are derived from the first 10 years and 71^th^~80^th^ years 1pctCO_2_ simulations, respectively. $d{\bar{C}}_{x,y}/d{\bar{T}}_{G}$, namely “pattern-change kernels” (PCKs), describes the shifts in ${\bar{C}}_{x,y}$ induced by human-forced climate warming. The implicit assumption here is that PCKs remain constant over the 70-year period interval. PCKs are constructed from the list of the 18 CMIP6 climate models (Table 2), with each climate model regridded to a 0.5° × 0.5° grid via area averaging (conservative regridding procedure) prior to PCKs construction. $\Delta {\bar{T}}_{G}$ is calculated as the difference between 10-year backward moving average of monthly global mean temperature from 2021 to 2100 and its corresponding 2012–2021 mean. ${\bar{V}}_{y}$ represents the monthly MESM zonal future climate from 2021 to 2100 and is calculated as the 20-year backward moving average of a particular month. For example, January 2021 is calculated as the average of January from 2002 to 2021. The 20-year moving average is intended to capture only the human-forced monotonic trend from MESM’s global climate response without introducing the internal climate variability to the baseline climate. ${V}_{x,y}$ represents the downscaled monthly future climate at the grid point (*x,y*) from 2021 to 2100. The monthly change (or “delta”) is the difference between ${V}_{x,y}$ and ${\bar{V}}_{x,y}^{{t}_{0}}$, which is further smoothed using a 21-year running average centered on each year for that month (the first and last 10 years have fewer samples for smoothing). We combine each of the 18 PCKs $\left(\frac{d{\bar{C}}_{x,y}}{d{\bar{T}}_{G}}\right)$ with the 50-member MESM zonal climate projections $\left({\bar{V}}_{y}\right)$ via the Eq. (2) to develop a 900-member ensemble per policy scenario. This meta-ensemble per scenario represents a comprehensive range of plausible outcomes resulting from the uncertainty in climate system response to anthropogenic emissions of greenhouse gases (50-member ensemble) and uncertainty in geographical patterns of climate change (PCKs).

## Data Records

Our global dataset comprises a 900-member ensemble of 9 climate variables (precipitation, mean, minimum and maximum air temperature, near-surface wind speed, shortwave and longwave radiation, specific humidity, and relative humidity) at 0.5° on a monthly time scale from 2021 to 2100 for four policy scenarios. Each ensemble member is packed in a single file of the self-describing netCDF format, a broadly accepted good-practice standard utilized in the weather and climate science research communities. The complete dataset is ~60 TB in size. All data are freely available at the repository of World Data Center for Climate (WDCC) at DKRZ^39^.

## Technical Validation

Below we provide an evaluation of this dataset that demonstrates the features of the detrended baseline climate, the emerging patterns extracted from the CMIP6 models, and the resultant regional distributions of climate changes across various scenarios. We adopt 21 regions^40^ to evaluate the dataset’s regional-scale performances. The detailed information of 21 regions is listed in Table 3. These regions represent different climatic regimes and physiographic settings, and are defined to approximately cover all land areas in the World with the size of each region varying in the range of a few thousand to several thousand km in each direction^40^. The evaluation is conducted on the regional averages of seasonal means across the entire period for each variable. The relevant ensemble statistics (minimum, median, and maximum) are derived based on the regional averages of seasonal means of each ensemble member. The evaluation is performed for all the meteorological variables and all the seasons [December-February (DJF), March-May (MAM), June-August (JJA), and September-November (NOV)] over the 21 regions from 1931 to 2010 (the detrended baseline) and from 2021 to 2100 (future climate) for four emission scenarios, respectively. Only precipitation and surface air temperature in DJF and JJA are included for demonstration purposes. Only land grid points are used, except for the global maps shown in Fig. 20.

**Table 3:** List of regions used in this study^40^. Only land grid points are used in the analysis.

Name	Acronym	Latitude (°)	Longitude(°)
Australia	AUS	45S-11S	110E-155E
Amazon Basin	AMZ	20S-12N	82W-34W
Southern South America	SSA	56S-20S	76W-40W
Central America	CAM	10N-30N	116W-83W
Western North America	WNA	30N-60N	130W-103W
Central North America	CNA	30N-50N	103W-85W
Eastern North America	ENA	25N-50N	85W-60W
Alaska	ALA	60N-72N	170W-103W
Greenland	GRL	50N-85N	103W-10W
Mediterranean Basin	MED	30N-48N	10W-40E
Northern Europe	NEU	48N-75N	10W-40E
Western Africa	WAF	12S-18N	20W-22E
Eastern Africa	EAF	12S-18N	22E-52E
Southern Africa	SAF	35S-12S	10W-52E
Sahara	SAH	18N-30N	20W-65E
Southeast Asia	SEA	11S-20N	95E-155E
East Asia	EAS	20N-50N	100E-145E
South Asia	SAS	5N-30N	65E-100E
Central Asia	CAS	30N-50N	40E-75E
Tibet	TIB	30N-50N	75E-100E
North Asia	NAS	50N-70N	40E-180E

### Seasonal PCKs

**Fig. 4:**
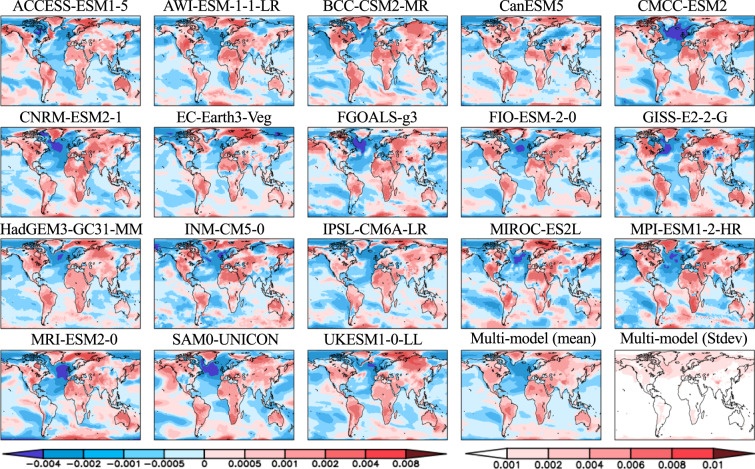
Global maps of the JJA averaged pattern-change kernels (PCKs) that describe the relative change in surface air temperature per unit change in global averaged surface-air temperature (units of K^−1^) from each of the 18 participating models in the Coupled Model Intercomparison Project Phase 6 (CMIP6) as well as the multi-model mean and standard deviation. PCKs are derived for each month from the 1% transient CO_2_ experimental simulations.

**Fig. 5:**
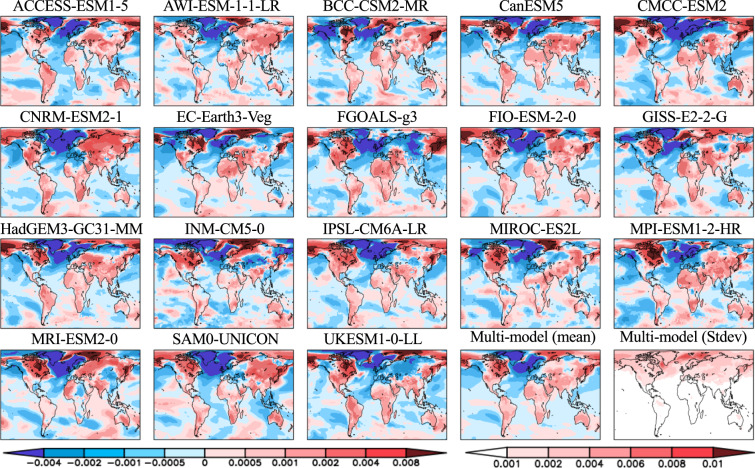
Same as Fig. 4, but for the DJF averaged pattern-change kernels (PCKs).

**Fig. 6:**
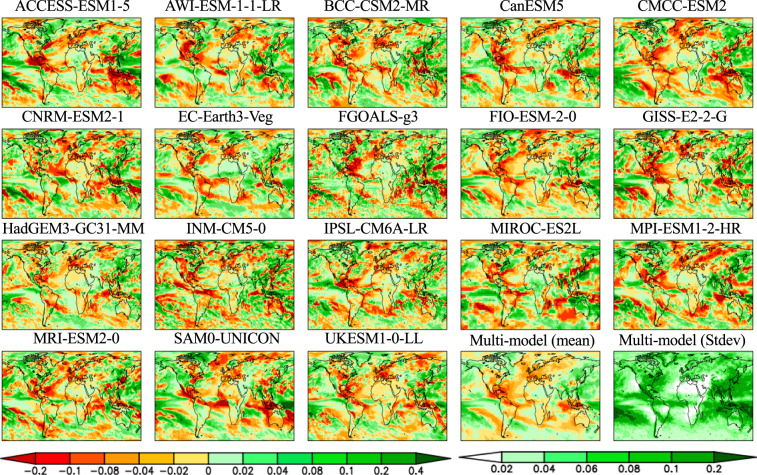
Global maps of the JJA averaged pattern-change kernels (PCKs) that describe the relative change in precipitation per unit change in global averaged surface-air temperature (units of K^−1^) from each of the 18 participating models in the Coupled Model Intercomparison Project Phase 6 (CMIP6) as well as the multi-model mean and standard deviation. PCKs are derived for each month from the 1% transient CO_2_ experimental simulations.

**Fig. 7:**
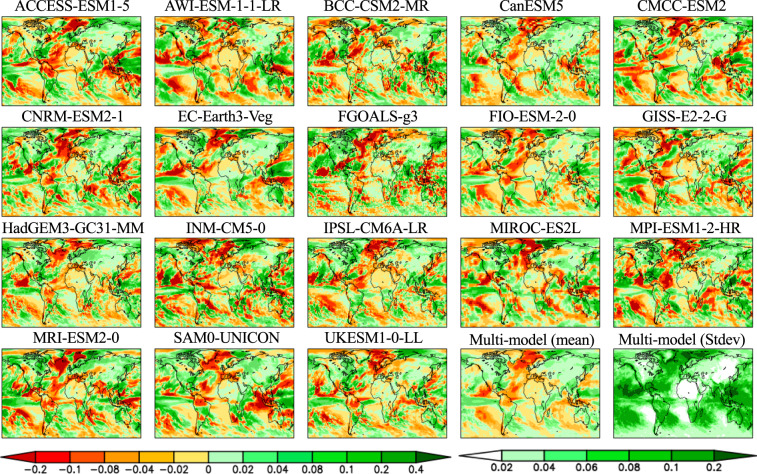
Same as Fig. 6, but for the DJF averaged pattern-change kernels (PCKs).

Seasonal PCKs of temperature and precipitation show a wide range of climate model response patterns as a result of forced climate warming (Figs. 4–7). The differences are reflected in the sign, magnitude, location, and extent of the changes in response. For temperature (Figs. 4, 5), the most striking feature is a colder ocean and warmer land (COWL) global pattern^41^ seen in both seasons across all the models, with the exceptions to this characterization found in different regions across various models. The model mean PCKs show that the relative cooling signal in land areas lies mostly over the northwesternmost regions of North America, western Europe, northern Siberia and parts of the southern Russia in DJF (Fig. 5). The maritime fetch of the relatively cooler ocean conditions may play a large role in the afore-mentioned relative cooling over the coastal regions. Also evident is the stronger warming over the high Northern Hemisphere latitudes in DJF than in JJA. High inter-model scatter in PCKs is ubiquitously observed in the boreal region of the Northern Hemisphere, particularly in DJF. In JJA, South Africa and interior portions of South America also present moderate inter-model scatter. The lowest model scatter generally occurs over much of the world’s oceans, particularly in the subtropics and with no evident seasonality. For precipitation (Figs. 6, 7), the model mean shows a wide extent of drier conditions attributed to climate warming, including a swath extending from central subtropical North Pacific to the North Atlantic and a path extending from the South Pacific basin to the South Atlantic and the Indian Ocean. The drying feature in these regions persists in both seasons, with the areas of prominent drying varying between two seasons. The drying is particularly enhanced over central subtropical North Pacific and the North Atlantic in DJF, but it is more prominent over the central America, Gulf of Mexico, Caribbean Sea and islands, east Indian Ocean and Indonesia islands in JJA. Over land areas, the most prominent drying occurs over Mexico, northern South America, northern African, and parts of the Middle East in DJF, but is largely confined to the Continental United States, Amazon, Southern Africa, Australia, the European continent, and Western Russia in JJA. The wetter conditions in both seasons over land areas are largely associated with the monsoon regions, including South and Southeast Asia, Africa, Australia, South and North America. High model scatter for precipitation is largely confined to the world’s oceans, particularly in DJF, including the subtropical Pacific, the western boundary of the North Atlantic, the tropical Atlantic, and the Indian Ocean. Over land, the Indian and Southeast Asian monsoon regions present the largest model scatter. Other notable areas with moderate model scatter include the eastern half of North America, most of Central America, South America, parts of Africa, Australia, Europe, and Western Russia. The lowest inter-model deviations occur in a large portion of Eurasia, Central North America, and Northern Africa in DJF, but are confined mostly in Northeast Brazil, Africa, and the Middle East in JJA.

### Detrended baseline climate

**Fig. 8:**
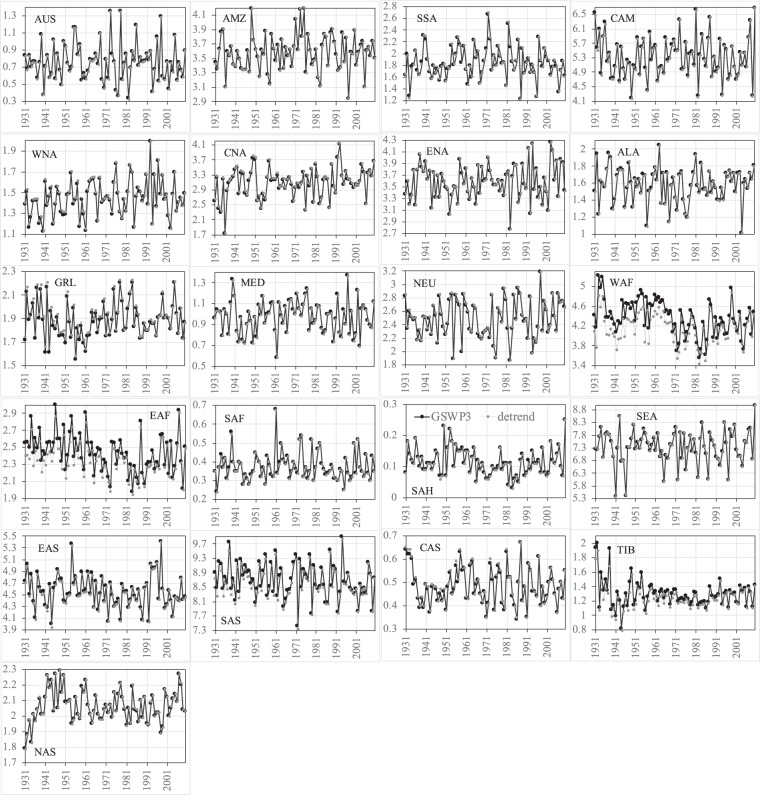
Comparisons between the GSWP3 (black line) and detrended baseline (gray line) JJA precipitation (mm/day) from 1931 to 2010 over the 21 regions.

**Fig. 9:**
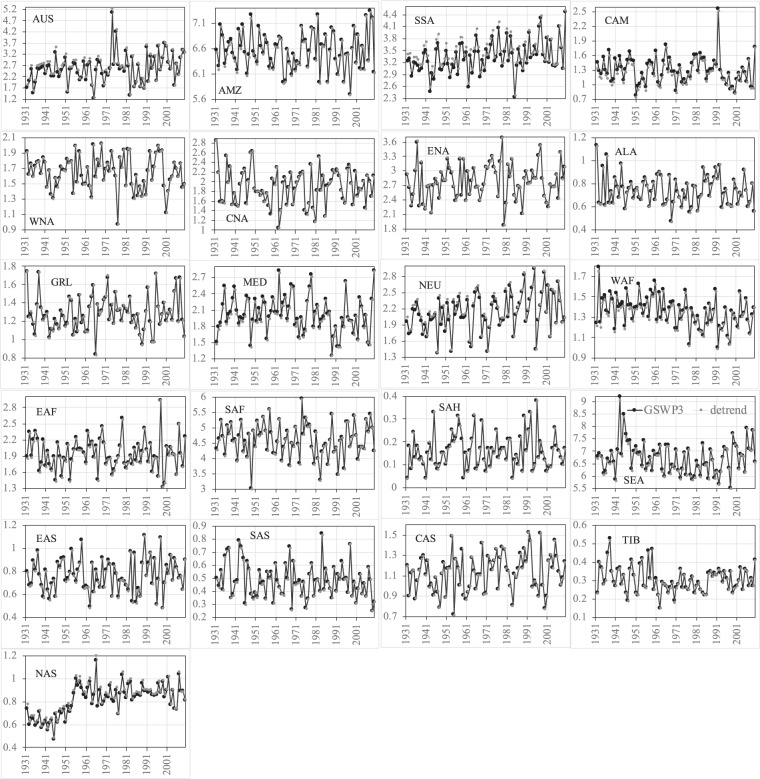
Comparisons between the GSWP3 (black line) and detrended baseline (gray line) DJF precipitation (mm/day) from 1931 to 2010 over the 21 regions.

**Fig. 10:**
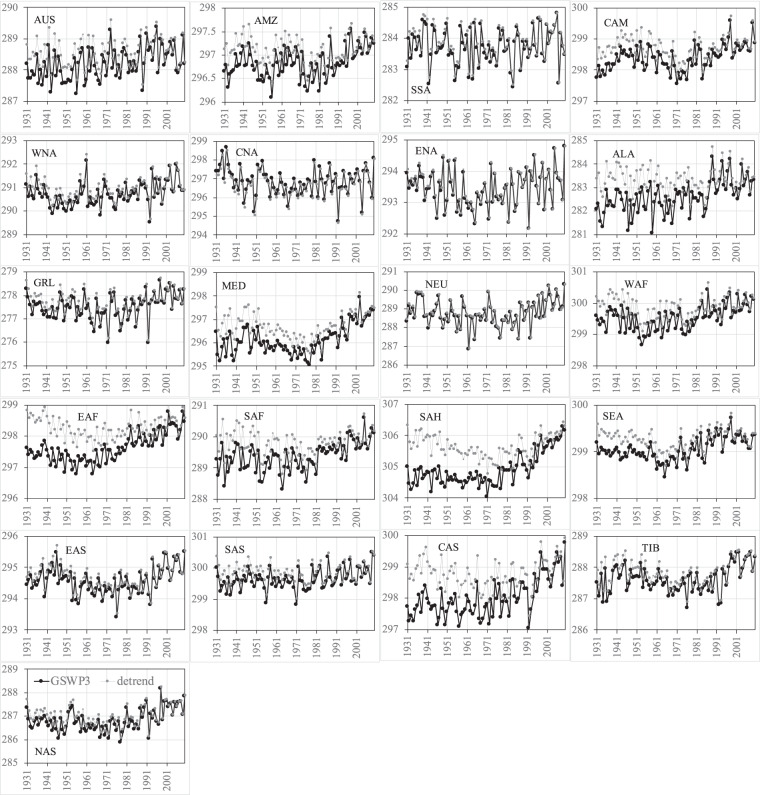
Comparisons between the GSWP3 (black line) and detrended baseline (gray line) JJA near-surface air temperature (K) from 1931 to 2010 over the 21 regions.

**Fig. 11:**
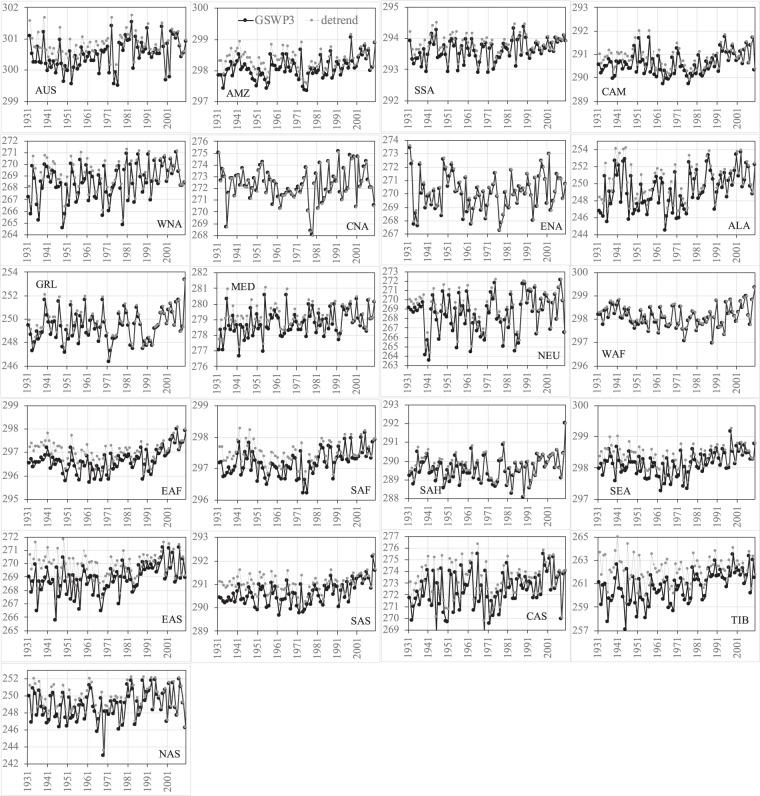
Comparisons between the GSWP3 (black line) and detrended baseline (gray line) DJF near-surface air temperature (K) from 1931 to 2010 over the 21 regions.

Figures 8–11 show the comparisons of precipitation and surface air temperature between GSWP3 and detrended baseline from 1931 to 2010 over the 21 regions. Overall, both JJA and DJF GSWP3 precipitation does not show evident trends over all the regions, except for the JJA precipitation in WAF and EAF (Figs. 8, 9). We see that the corresponding detrended baseline precipitation eliminates or reduces the trends in these two regions. Detrending is more clearly observed in surface air temperature (Figs. 10, 11). As expected, GSWP3 surface air temperature exhibits strong upward trends over a majority of regions for both seasons. Some exceptions are observed for SSA, North America (WNA, CNA, ENA), GRL, and NEU in both seasons, for Asia (except for CAS) and TIB in JJA, as well as for WAF, SAH, and NAS in DJF. GSWP3 surface air temperature in DJF presents overall weaker upward trends than in JJA, except for EAS, SAS, and TIB. Regions in the same continent may display very different patterns. For example, CAS is only one out of four regions in Asia showing a strong upward trend in JJA temperature. Overall, the detrended baseline climate is seen to eliminate or reduce the trends inherent in the GSWP dataset to varying degrees, regardless of the meteorological variables, seasons, and regions.

### Future climate

**Fig. 12:**
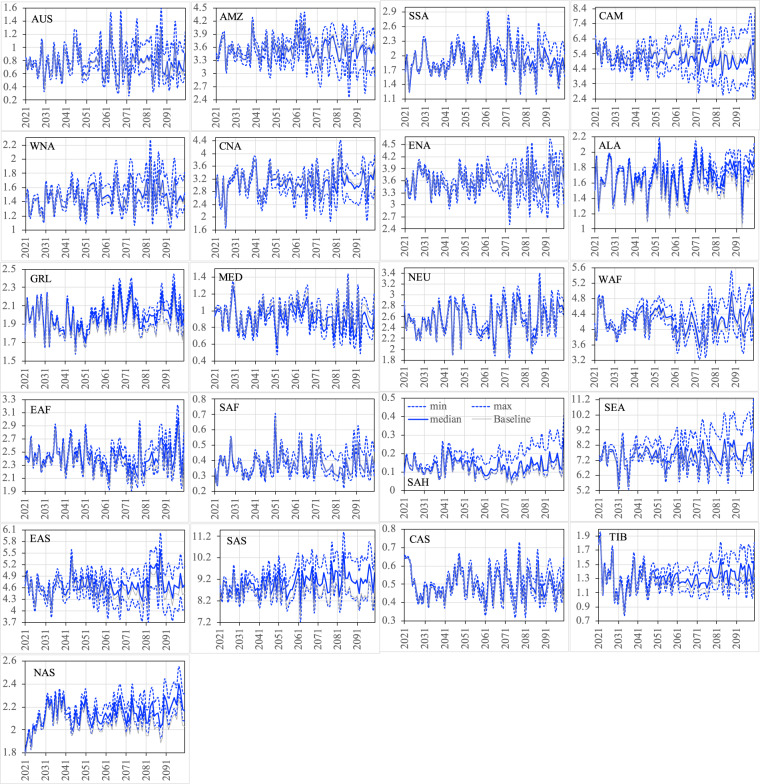
The ensemble statistics (minimum, median, and maximum) of JJA precipitation (mm/day) from 2021 to 2100 over the 21 regions for the PF scenario, along with their detrended baseline counterparts.

**Fig. 13:**
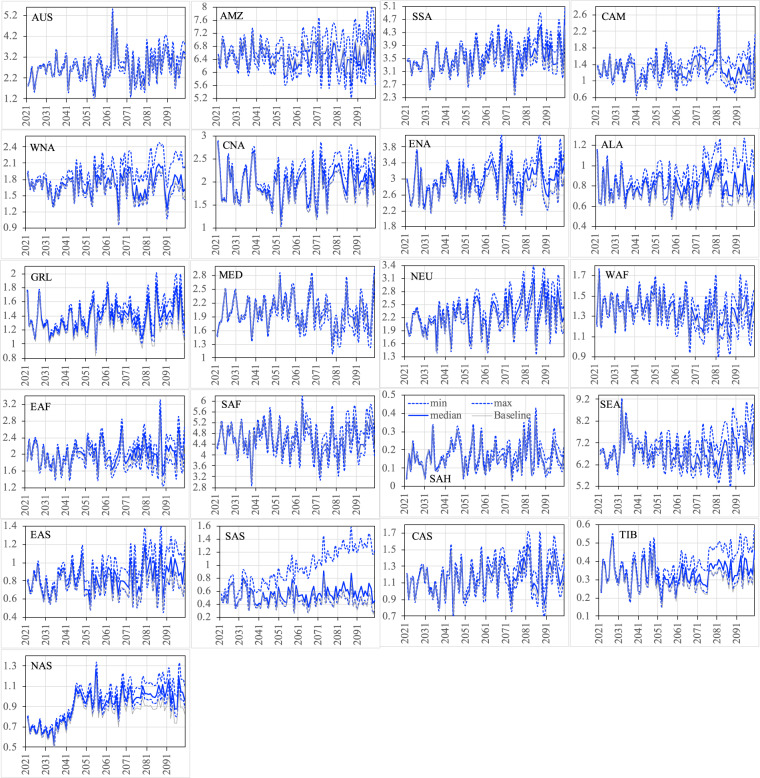
The ensemble statistics (minimum, median, and maximum) of DJF precipitation (mm/day) from 2021 to 2100 over the 21 regions for the PF scenario, along with their detrended baseline counterparts.

**Fig. 14:**
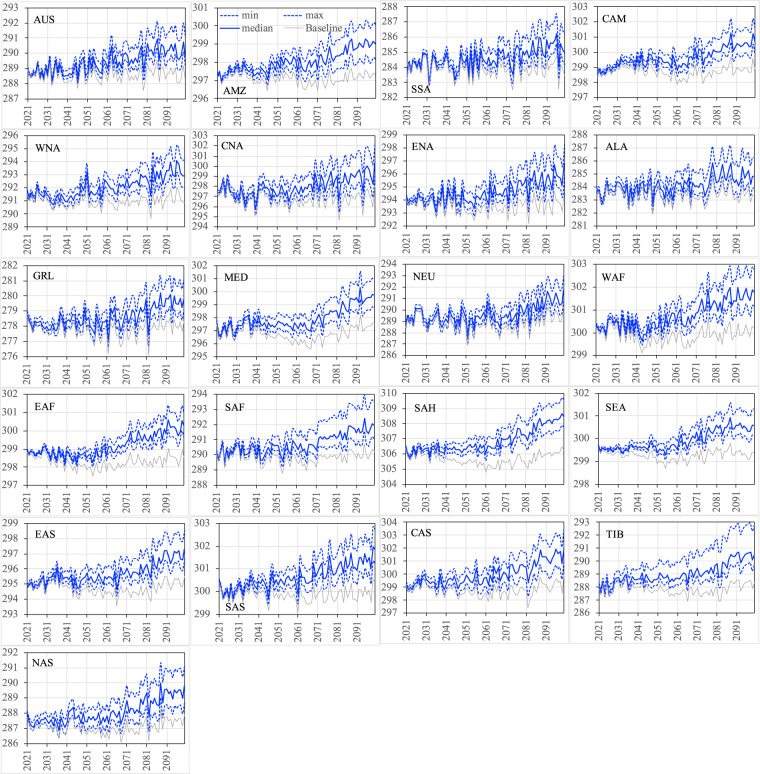
The ensemble statistics (minimum, median, and maximum) of JJA near-surface air temperature (K) from 2021 to 2100 over the 21 regions for the PF scenario, along with their detrended baseline counterparts.

**Fig. 15:**
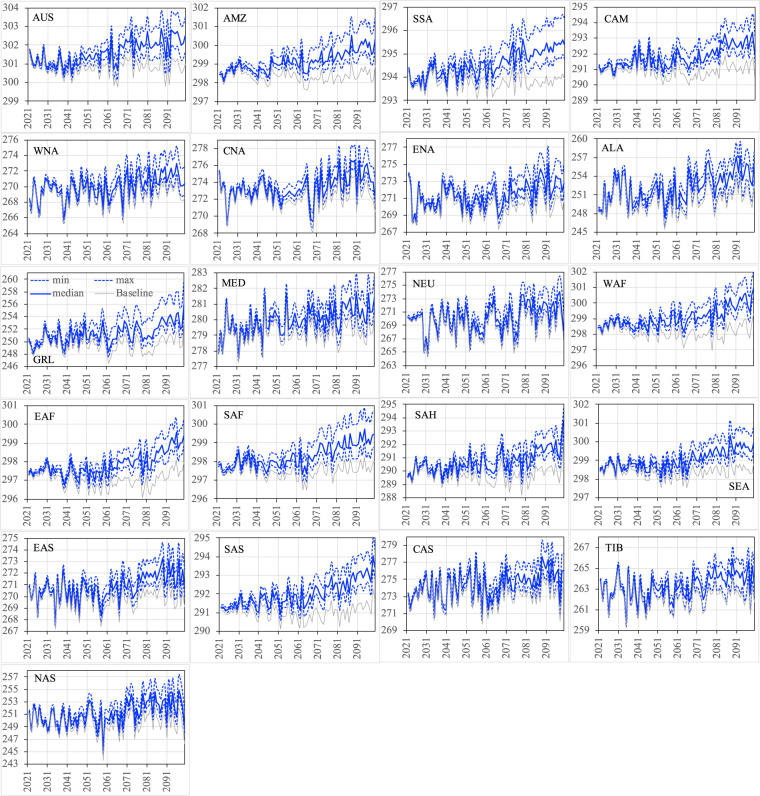
The ensemble statistics (minimum, median, and maximum) of DJF near-surface air temperature (K) from 2021 to 2100 over the 21 regions for the PF scenario, along with their detrended baseline counterparts.

Figures 12–15 show the ensemble statistics (minimum, median, and maximum) of precipitation and surface air temperature in both seasons from 2021 to 2100 for the PF scenario, along with their detrended baseline counterparts. Ensemble medians of precipitation are largely aligned with the detrended baseline over most regions (Figs. 12, 13), with some exceptions in the high latitudes (ALA, GRL, NEU, and NAS) and dry regions (EAF, SAH, and SAS in JJA as well as WNA, CNA, and ENA, EAS, SAS, and TIB in DJF). Ensemble uncertainty (spread) over all the regions is small at the beginning of the period and becomes increasingly larger by the end of the century. The high latitude and dry regions are typically characterized by the small ensemble uncertainty in both seasons. The large ensemble uncertainty is generally found around the tropics, such as CAM (3.66 mm/day) and SEA (2.87 mm/day) in JJA as well as SEA (1.54 mm/day) and AMZ (1.18 mm/day) in DJF, consistent with what was found in previous studies^42–45^. All the ensemble members of surface air temperature exhibit consistently higher values than the detrended baseline throughout the entire period in both seasons over all the regions (Figs. 14, 15). The tropical regions present an overall small ensemble uncertainty in both seasons. The ensemble spread of JJA air temperature varies from 1.13 °C in the SEA to 3.16 °C in the CNA by 2100. TIB is the only other region with the ensemble uncertainty of JJA air temperature exceeding 3 °C (3.02 °C). DJF air temperature features large ensemble uncertainty in the high latitudes, particularly in the GRL (4.7 °C) and ALA (4.9 °C) regions, and captures the influence of the heterogeneity in the representations of snow and cold land processes by various Earth-system models^46,47^. These patterns of temperature uncertainty agree with what was shown in the CMIP6 models^45^.

**Fig. 16:**
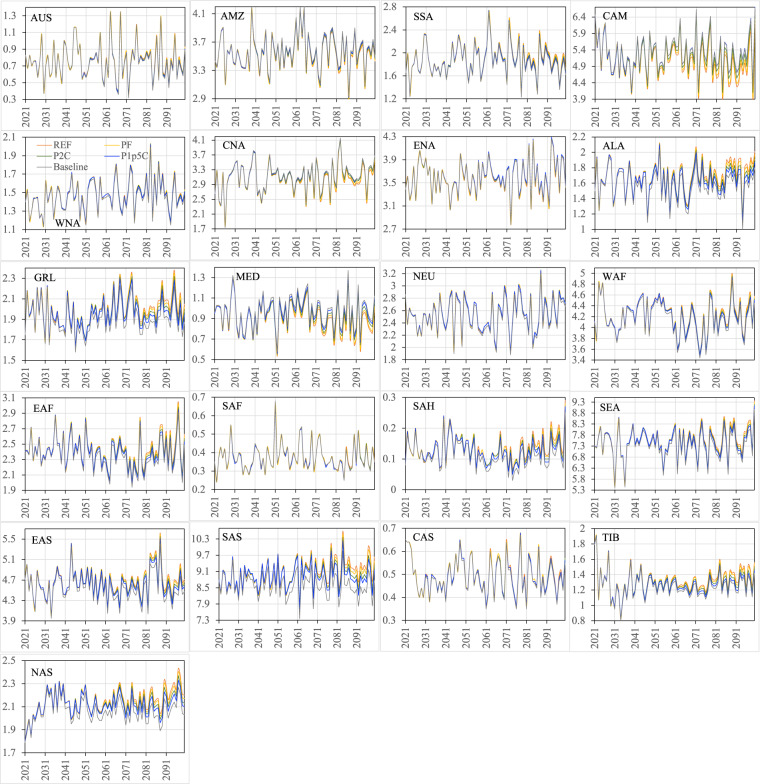
The ensemble medians of JJA precipitation (mm/day) from 2021 to 2100 over the 21 regions for all the four policy scenarios, along with their detrended baseline counterparts.

**Fig. 17:**
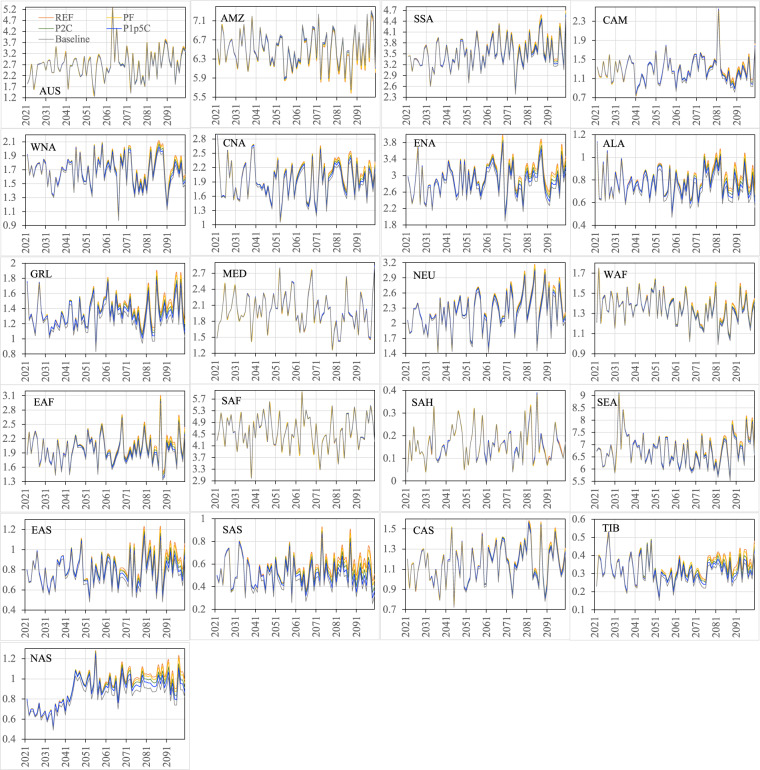
The ensemble medians of DJF precipitation (mm/day) from 2021 to 2100 over the 21 regions for all the four policy scenarios, along with their detrended baseline counterparts.

**Fig. 18:**
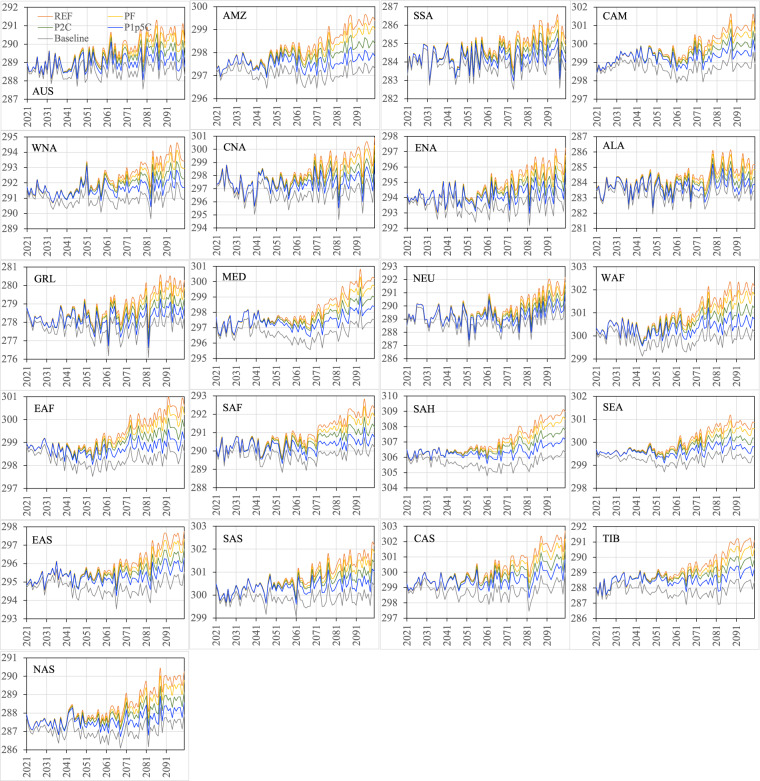
The ensemble medians of JJA near-surface air temperature (K) from 2021 to 2100 over the 21 regions for all the four policy scenarios, along with their detrended baseline counterparts.

**Fig. 19:**
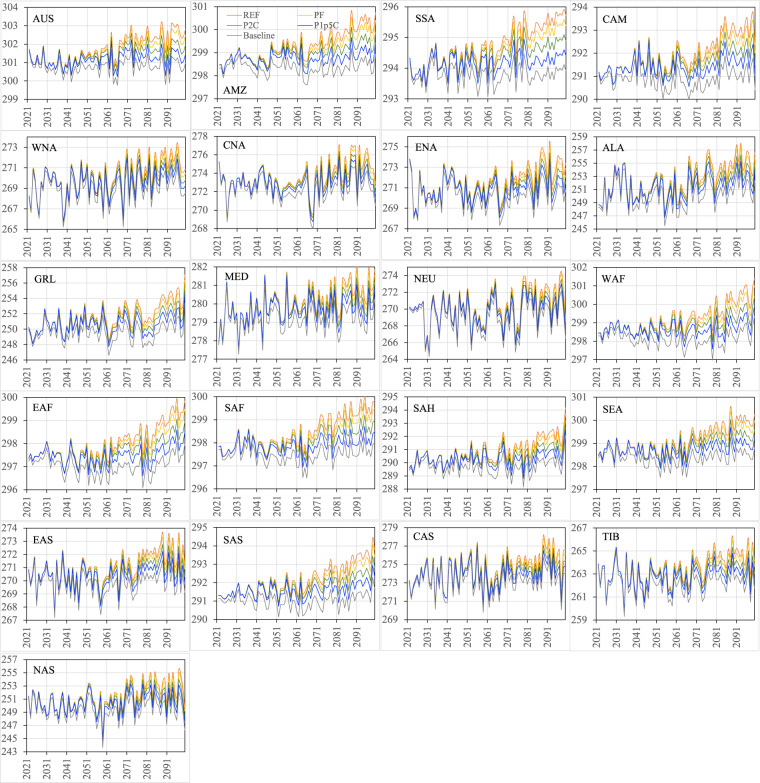
The ensemble medians of DJF near-surface air temperature (K) from 2021 to 2100 over the 21 regions for all the four policy scenarios, along with their detrended baseline counterparts.

Figures 16–19 show the ensemble medians of precipitation and surface air temperature in both seasons from 2021 to 2100 for all the four scenarios, along with their detrended baseline counterparts. The differences in precipitation of both seasons (Figs. 16, 17) attributed to the scenarios are much smaller than those attributed to the ensembles of the specific scenario (e.g., PF in Figs. 12, 13). By 2100, the spread of JJA precipitation across four scenarios (the difference between REF and P1p5C) is less than 0.2 mm/day over most of the regions (Fig. 16) and the spread in DJF precipitation is even smaller (Fig. 17). The dominance of model uncertainty over scenario uncertainty in regional precipitation projection uncertainty was also reported in previous studies^45,48^. Regardless of the scenario, precipitation of both seasons does not exhibit apparent drying or wetting trend over all the regions. In contrast, climate policy (P1p5C) effectively reduces surface air temperature, with the reduction ranging from 1.1 °C to 1.9 °C in JJA (Fig. 18) and from 1.0 °C to 2.9 °C in DJF (Fig. 19). The strongest reduction in JJA air temperature occurs mostly in the mid latitudes (e.g., CNA, CAS, MED, TIB) and SAH desert, while that in DJF is found mostly in high latitudes (2.9 °C in ALA, 2.5 °C in GRL, 2.4 °C in NAS, 1.9 °C in ENA). Many studies documented “Arctic amplification”, a phenomenon that the warming in the Arctic far outpaces the global average^49,50^, particularly in winter^51^. Climate mitigation efforts could thus have significant implications on land ice loss, wildlife and human livelihoods, and methane emissions in these high latitude regions.

**Fig. 20:**
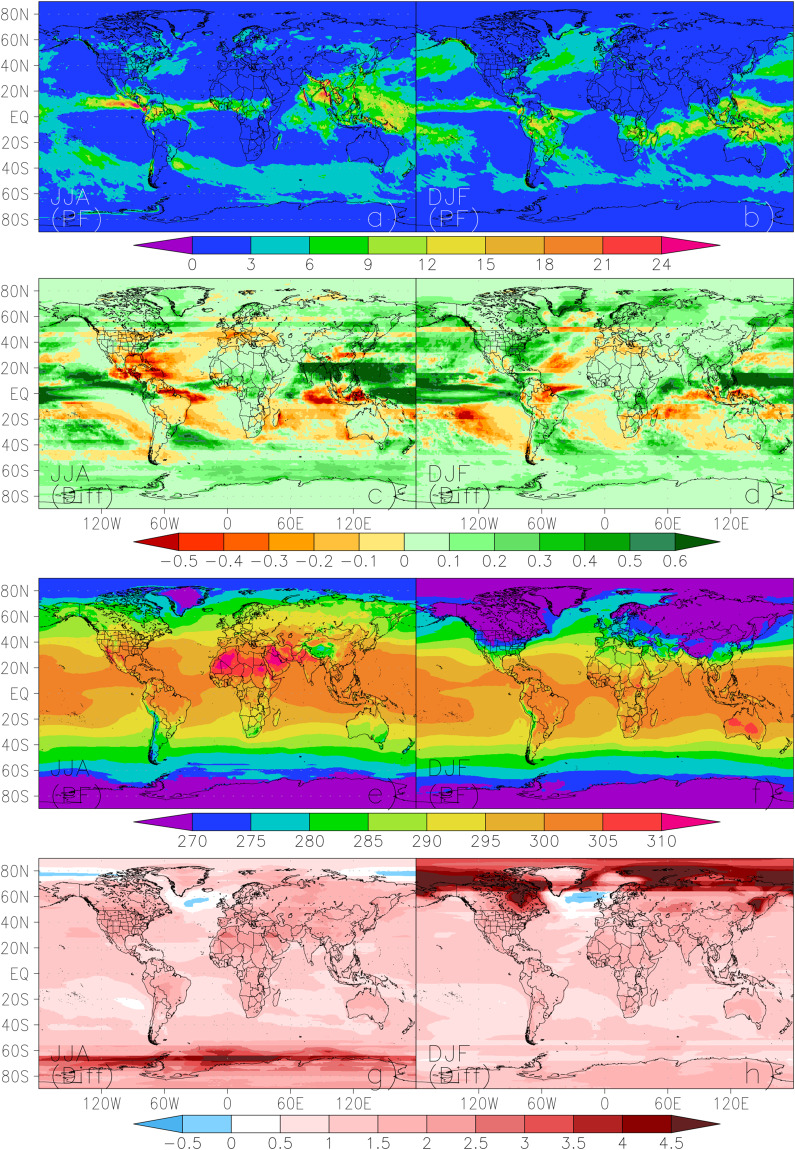
Ensemble medians of the precipitation (panels a-d, mm/day) and surface air temperature (panels e-h, K) in JJA (left columns) and DJF (right columns) in 2090 for the PF scenario, along with the differences (panels c,d, g, and h) from its corresponding detrended baseline counterparts in 2000.

Figure 20 presents the ensemble medians of precipitation and surface air temperature in both seasons in 2090 for the PF scenario, along with the differences from its corresponding detrended baseline counterparts. The ensemble median precipitation in both seasons indicate that maxima occur in the Tropics, specifically the intertropical convergence zone (ITCZ) in the Atlantic, Pacific, and Indian Oceans, the South Pacific convergence zone (SPCZ), as well as over tropical Africa and South America (Fig. 20a,b). This band of heavy rain moves north and south of the Equator seasonally. Major precipitation peaks in tropical regions are below the equator in DJF but are located further north in JJA. JJA is the season that is strongly affected by the Asian monsoon which brings heavy rain to China, southeast Asia, and India. In midlatitudes, the storm tracks in the Northern Hemisphere (NH) oceans are much stronger in DJF than in JJA, while the circumpolar storm tracks in the Southern Hemisphere (SH) are weaker in DJF than in JJA. DJF is also characterized by a secondary precipitation maximum along the northwest coast of North America from Alaska to California at the eastern end of the Pacific Ocean storm track. Except for the Maritime Continent, a majority of land areas are characterized by dry condition in both seasons with moderate precipitation found in Eastern North America, northern Europe, northeastern and southeastern China in JJA. These characteristics confirm the well-established behaviors from climate models in these regions.

Many regional characteristics of wetting and drying in the ensemble median precipitation changes are shared by both seasons and consistent with theoretical expectations and/or findings revealed in previous studies^52^ (Fig. 20c,d). The strongest positive changes are found in the equatorial Pacific with many regions being associated with Monsoon precipitation and they shift slightly between north and south seasonally. Extensive but weaker positive changes are also observed in the high latitudes (lands and oceans) of both hemispheres. A strong drying signal is projected over the central America, the Amazon, the Mediterranean, and Indonesia islands together with the Atlantic, Indian, and South Pacific Oceans. The most part of the United States (US) is projected to become wetter in DJF, particularly in the Eastern US and the west coast, while a large portion of the US will experience precipitation decreases in JJA, including the northern part of the country and the South-Central US. In-between lies a moderate wetting zone extending from the west to the east.

The ensemble medians of JJA and DJF surface air temperature (Fig. 20e,f) demonstrate the well-established temperature gradient from the equator to the high latitudes of both hemispheres. The JJA temperature maxima are located in the Sahara Desert, the Middle East, the Mojave Desert, and the Sonoran Desert in northwestern Mexico, while the temperature minima are confined to the Greenland and Antarctic. DJF temperature maxima are found in the Australian Deserts, while the minima are concentrated in the high latitude lands (North of 40°N) and Antarctic. The ensemble median air temperature is projected to increase over most of the globe relative to the detrended baseline, but the magnitude of increase varies regionally and seasonally (Fig. 20g,h). The immediately evident are the strongest JJA warming in the Antarctic Peninsula and the strongest DJF warming in the northern high latitudes (in particular the Arctic regions). Previous studies reported that the Arctic regions experience warming at twice the pace of the global average^49,50^ and unprecedented warming over the Antarctic Peninsula^53^. The general pattern of temperature increase indicates that warming is stronger over land than over oceans. The limited cooling signals are found over the northern Atlantic in both seasons as well as over the Arctic Ocean in JJA. Previous studies linked the cooling in the northern Atlantic to the slowing of the Atlantic Meridional Overturning circulation and the resulting slower northward surface-heat transport^54,55^. These pattern characteristics are consistent with what was shown in the CMIP6 models^52^.

In general, the evaluation suggests that the dataset is able to capture broadly the well-observed climate features across various regions of the globe and the results are consistent with what previous studies reported. This meta-ensemble, high-resolution, bias-corrected global dataset of long-term future climate can be used for meeting various needs associated with climate impact assessments, including uncertainty analyses, risk quantification, climate policy mitigation analyses, and driving climate impact models which require monthly data inputs, on both global and regional scales. Some caveats do exist, for example, the dataset cannot adequately represent the frequency and intensity of various extreme events (i.e., flooding, heatwave, or droughts) and users should thus take caution to use the data in this regard.

## Data Availability

All the relevant codes to produce the global dataset of future climates are publicly available at the GitHub repository (https://github.com/mit-jp/SD-BC). The pattern-change kernels” (PCKs) for all the CMIP6 models are created using Grid Analysis and Display System (GrADS). The SD-BC procedure is carried out using FORTRAN 90 programming language.
